# Probiotics and MicroRNA: Their Roles in the Host–Microbe Interactions

**DOI:** 10.3389/fmicb.2020.604462

**Published:** 2021-01-14

**Authors:** Ying Zhao, Yan Zeng, Dong Zeng, Hesong Wang, Mengjia Zhou, Ning Sun, Jinge Xin, Abdul Khalique, Danish Sharafat Rajput, Kangcheng Pan, Gang Shu, Bo Jing, Xueqin Ni

**Affiliations:** ^1^Animal Microecology Institute, College of Veterinary, Sichuan Agricultural University, Chengdu, China; ^2^Guangdong Provincial Key Laboratory of Gastroenterology, Department of Gastroenterology, Institute of Gastroenterology of Guangdong Province, Nanfang Hospital, Southern Medical University, Guangzhou, China; ^3^Sichuan Academy of Animal Sciences, Animal Breeding and Genetics Key Laboratory of Sichuan Province, Chengdu, China

**Keywords:** probiotics, microRNA, gut microbiota, homeostasis, host–microbe interactions

## Abstract

Probiotics are widely accepted to be beneficial for the maintenance of the gut homeostasis – the dynamic and healthy interactions between host and gut microorganisms. In addition, emerging as a key molecule of inter-domain communication, microRNAs (miRNAs) can also mediate the host–microbe interactions. However, a comprehensive description and summary of the association between miRNAs and probiotics have not been reported yet. In this review, we have discussed the roles of probiotics and miRNAs in host–microbe interactions and proposed the association of probiotics with altered miRNAs in various intestinal diseases and potential molecular mechanisms underlying the action of probiotics. Furthermore, we provided a perspective of probiotics–miRNA–host/gut microbiota axis applied in search of disease management highly associated with the gut microbiome, which will potentially prove to be beneficial for future studies.

## Introduction

Trillions of microbes exist in the human gastrointestinal tract, and the number of these microbes greatly exceeds the number of human body cells, which influences the physiological activities of the host throughout the lifespan (Li D. et al., 2016; Chang and Kao, 2019). This complicated ecosystem containing bacteria, fungi, viruses, and protists is called the gut microbiota. Intense exploration for understanding the relationships between host and gut microbiota has never ceased. In particular, the “dysbiosis” of gut microbiota may lead to the occurrence of various diseases of the host, ultimately damaging its health (Li D. et al., 2016; Chang and Kao, 2019). Probiotics, in sufficient doses, can restore the intestinal microecological balance and promote the healthy growth of the host (Hill et al., 2014). The consumption of probiotics is prevalent because they have the potential to become an important intervention strategy to prevent and treat various diseases (Balakrishnan and Floch, 2012; Suez et al., 2019; Yousefi et al., 2019). Although probiotics have great prospects, concerns about security and doubts about their effectiveness are always present. It is important to explain the exact molecular mechanism describing probiotics’ influence on host–microbe interactions, which remains largely unknown so far. MicroRNAs (miRNAs) are considered to be important regulators in the host gene expression process, and some are found to be significantly related to the microbial community or specific bacteria (Hasan and Yang, 2019). Recent studies have proposed the functional role of miRNAs played in shaping gut microbiota (Moloney et al., 2019; Behrouzi et al., 2020). These host-derived miRNAs were able to enter bacterial cells to cultivate or inhibit specific bacteria (Liu et al., 2016; Spinler et al., 2019). These effects on host–microbe interactions suggested that miRNAs may be important participants in the molecular mechanism of probiotics action.

In this review, we first summarized the interactions between host and gut microbiota and presented a brief overview of probiotic functions and their impact on host–microbe interactions. Then, we highlighted the importance of miRNAs to host gastrointestinal function and described the correlation between miRNA expression levels and gut microbiota profiles. Next, we discussed the current understanding of the host’s use of fecal miRNA and plant-derived miRNA in maintaining gut homeostasis. Finally, we proposed the potential molecular mechanism underlying how probiotics maintain gut homeostasis and proposed the concept of probiotics–miRNA–host/gut microbiota axis that can serve as a new direction for future exploration.

## Interactions Between Host and Gut Microbiota

Genes encoded by the gut microbiota are 150 times larger than the human genome, which includes an extremely rich enzyme repository (El Kaoutari et al., 2013; Rooks and Garrett, 2016; Rowland et al., 2018; Zimmermann et al., 2019). Most members of gut microbiota exert harmless or beneficial effects on the host. They provide uncoded human enzymes, participate in human metabolism, and also maintain immune homeostasis through their interactions with host cells (Rooks and Garrett, 2016; Rowland et al., 2018). For example, the bacterial fermentation products of carbohydrates are short-chain fatty acids (SCFAs) that are important energy sources for bacteria itself and host cells (Rowland et al., 2018). SCFAs also have varying regulatory functions on the host, including the enhancement of epithelial barrier function and maintenance of mucosal immunity (Rooks and Garrett, 2016; Rowland et al., 2018). Additionally, bile acids are classical examples of the interactions between host and gut microbiota. The gut microbes modified the bile acids synthesized in the liver into secondary bile acids that modulate multiple host metabolic processes and immune homeostasis (Long et al., 2017; Parasar et al., 2019). In contrast, bile acids are capable of influencing the growth of bacteria, resulting in changes in the structure of the microbial community. The gut microbiota is essential for host metabolism and immune homeostasis, and their components contribute considerably to shaping the host immune system, such as lipopolysaccharide, flagellin, peptidoglycan, etc. (Rooks and Garrett, 2016).

**FIGURE 1:**
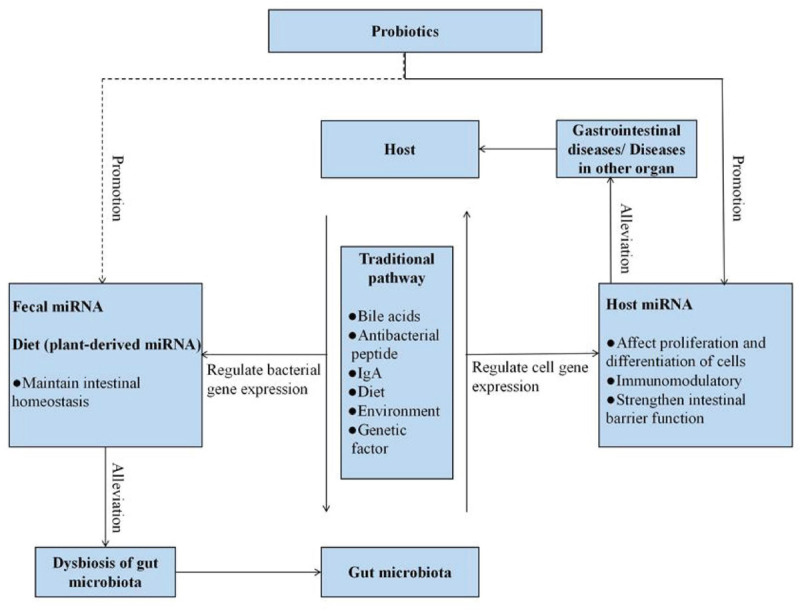
Probiotics and miRNA, as well as their implications for host–microbe interactions. The traditional pathways involving in host–microbe interactions are mainly dominated by bile acids, antibacterial peptide, and IgA. In addition, the traditional pathways also include dietary, environmental, and genetic factors. Moreover, the host regulates bacterial gene expression *via* fecal miRNA and plant-derived miRNA to shape gut microbiota. Gut microbiota regulate cell gene expression *via* host miRNA to alleviate various gastrointestinal diseases. Probiotics have impacts on these host–microbe interactions. The dotted line represents the lack of conclusive evidence in the relationship at present.

Interactions between the host and gut microbiota promote the establishment of a symbiotic relationship during host development. The optimal operation of a symbiotic relationship is vital to host health. But, the performance of gut microbiota is affected by various factors, including diet, lifestyle, illness, environment, hygiene, genetics, and antibiotic exposure (Li D. et al., 2016; Cristofori et al., 2018; Celiker and Kalkan, 2020). Although the composition of specific flora may change due to the stimulation of internal and external factors, the structure of the intestinal microbial community remains dynamic and stable (Coyte et al., 2015). Differences in the types of microorganisms are carried by different individuals, but the composition and function of the “core microbiome” are similar and remain relatively stable over time (Lozupone et al., 2012; Coyte et al., 2015; Milani et al., 2017; Gentile and Weir, 2018). This microbial stability is considered to play a critical role in the health of the host. It is worth mentioning that it is regarded as a sign of host intestinal health. Disruption of microbial stability often leads to “dysbiosis,” whichinduces a range of diseases (Lozupone et al., 2012; Coyte et al., 2015; Li D. et al., 2016; Milani et al., 2017). Moreover, gut microbiota have the ability to trigger responses from a distance by producing metabolites (Agus et al., 2018; Allaire et al., 2018; Gentile and Weir, 2018). The crosstalk between host and gut microbes can be linked to the health status of other body organs (Gresse et al., 2017; Agus et al., 2018; Allaire et al., 2018; Gentile and Weir, 2018; Adak and Khan, 2019; Maslowski, 2019; Zhang et al., 2019). Another aspect of the crosstalk includes the detailed mechanism of host manipulation of the bacterial community that has also received extensive attention (Figure 1). The traditional pathways are mainly dominated by bile acids, antibacterial peptides, and IgA (Brown et al., 2013; Chu and Mazmanian, 2013; Liu, 2016; Parker et al., 2018). Additionally, compelling evidences supported that genetic and environmental factors contributed greatly to the occurrence of dysbiosis, which in turn promoted many diseases, such as obesity and inflammatory bowel disease (IBD; Zhou M. et al., 2017; Cuevas-Sierra et al., 2019; Celiker and Kalkan, 2020). Recent studies have proposed a novel mechanism that the host specifically controls the gut microbiota by promoting the liberation of miRNAs (Liu et al., 2016). Diet and probiotics are also valuable tools available to alter the gut microbiota (Forgie et al., 2019; Tang, 2019).

## Probiotics and Their Implications for Host–Microbe Interactions

**FIGURE 2:**
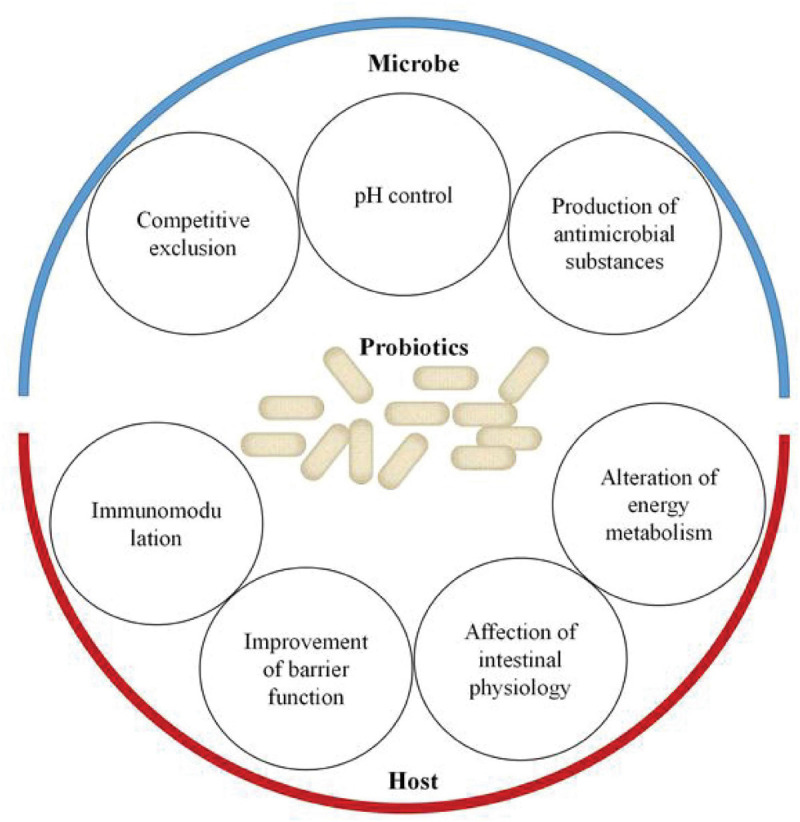
Effects of probiotics on the host–microbe interactions. Probiotics support the stability of the bacterial community and restore the “signature” of gut microbiota through competitive exclusion, pH control, and production of antimicrobial substances. On the other hand, probiotics promote health benefits for the host through improvement of barrier function, immunomodulation, affection of intestinal physiology, and alteration of energy metabolism.

Probiotics are commercial food supplements that are widely used all over the world. It has been generally accepted that they are able to restore the disruption of gut microorganisms. In 2002, probiotics were defined by WHO as live microorganisms that confer a health benefit when administered in adequate amounts (Hill et al., 2014). At present, as shown in Figure 2, various methods, including metagenomics, transcriptomics, and metabolomics, have already been applied to describe the mechanisms of the effects of probiotics on the host–microbe interactions (Bermudez-Brito et al., 2019; Kiousi et al., 2019; Yadav and Pratyoosh, 2019; Quigley and Gajula, 2020; Sehrawat et al., 2020). The most commonly beneficial effect of the probiotic administration route is to stabilize the bacterial community and restore the “signature” of gut microbiota. It mainly stems from their ability to produce bacteriocins, lower pH, and compete with pathogens for space and nutrients (Balakrishnan and Floch, 2012; Cremon et al., 2018; Suez et al., 2019). Competitive exclusion by probiotics is based on the competition for available nutrients and adhesion sites. It has been demonstrated that purified surface adhesion proteins of probiotics can exclude pathogens. Surface adhesins of pathogens and probiotic strains are implicated in mediating the binding of microbes to the host (Du et al., 2010). Probiotics can also change the environment to gain a more favorable competitive advantage. Studies on lactobacilli and bifidobacteria have shown that these two probiotic strains could produce organic acids to lower intestinal pH. Both organic acids and bacteriocins produced by bacteria enable the inhibition of pathogen growth (Dobson et al., 2012). On the other hand, probiotic bacteria (e.g., *Lactobacillus* and *Bifidobacterium*) have been revealed to improve barrier function. The ability of probiotics to upregulate expression levels of mucus-secretion genes may be one of the mechanisms of improving barrier function and excluding pathogens (Khoruts, 2018; Bermudez-Brito et al., 2019; Sanders et al., 2019; Yan and Polk, 2020). Furthermore, probiotic strains have been reported to directly exert immune and anti-inflammatory effects on the host. Probiotics may affect the immune system by changing the levels of metabolites, components, and DNA. They have been shown to activate immune cells, increase the production of immunoglobulins, and regulate cytokines (Khoruts, 2018; Sanders et al., 2019; Liwinski and Elinav, 2020; Yan and Polk, 2020). Several studies have demonstrated that these probiotic-derived factors can suppress intestinal inflammation *via* targeting toll-like receptor (TLR) signaling (Gómez-Llorente et al., 2010). Additionally, probiotics could also alter energy metabolism and impact intestinal physiology by releasing metabolites including the SCFAs, oligosaccharides, vitamins, amino acids, and secondary bile acids (Gura, 2014; Lee et al., 2018; Bermudez-Brito et al., 2019; Kiousi et al., 2019; Quigley and Gajula, 2020; Sehrawat et al., 2020). For instance, a recent study found that the SCFAs produced by *Lactobacillus casei* and *Bifidobacterium breve* could potentially be an essential regulatory effector of intestinal epithelial cell (IEC) proliferation (Matsuki et al., 2013). Currently, nutritional programs have received increasing attention; probiotic administration manipulates the gut microbiota to prevent or attenuate metabolic-related diseases (e.g., hypertension and hyperglycemia) (LeBlanc et al., 2017).

As probiotics can prevent and/or treat various diseases, their consumption is becoming more and more common all over the world. The growing industry shows that probiotics have a huge market prospect and the global market is expected to reach $65 billion by 2024 (Abid and Koh, 2019). Of note, both current research and application have found some limitations of probiotics. The action level of probiotics depends on different conditions, including the strains, individuals, types of administration, or location within the intestines (Markowiak and Śliżewska, 2017; Kothari et al., 2019; Langella and Chatel, 2019; Salvucci, 2019; Wan et al., 2019). Although compelling evidence has been obtained in clinical trials, the exact molecular mechanism of probiotics that confer beneficial effects on the host remains unclear. Of late, new possibilities have been observed in different studies exploring the interaction between miRNA and microbiota and the responses of miRNA to probiotic intervention.

## What Is miRNA?

MicroRNAs are endogenous, small, non-coding RNAs that are 19–25 nucleotides in length (Yu et al., 2016). They are first synthesized in the nucleus, transported into the cytoplasm, and processed into mature miRNAs (Yu et al., 2016; Bartel, 2018). Mature miRNAs are loaded onto the miRNA-induced silencing complex (miRISC) and bound to the 3’ untranslated region (3’ UTR) of mRNA to mediate post-transcriptional gene silencing (Yu et al., 2016; Bartel, 2018).

Despite complexity was added in terms of gene regulation, interest has surged in the function of miRNA molecules to mediate gene silencing to repress protein synthesis. Since the first miRNA, lin-4, was discovered in the 90s in *Caenorhabditis elegans*, hundreds of different miRNAs were found to play critical roles in healthy and pathological cell processes, including cellular differentiation, proliferation, signal transduction, and apoptosis (Fischer, 2015; Mohr and Mott, 2015; Yu et al., 2016; Rupaimoole and Slack, 2017; Bartel, 2018). Insights into the ability of miRNAs had made them into promising therapeutic tools (miRNA mimics) or targets (miRNA inhibitors) in cancer and other diseases (Link et al., 2010; Kalla et al., 2015; Rupaimoole and Slack, 2017; Tili et al., 2017; Hossian et al., 2019; Chen et al., 2020).

An early encouraging work demonstrated that miRNAs could be isolated from circulating body fluids (Weber et al., 2010; Lu and Rothenberg, 2018; Lee, 2019). They exist and transfer stably outside the cell and in feces through vesicles. Exosomes (30–100 nm nanosized vesicles) derived from multiple cell types are capable of encapsulating protein and RNA molecules (Turchinovich et al., 2011; Celluzzi and Masotti, 2016; Yu et al., 2016; Bartel, 2018; Moloney et al., 2019). miRNA molecules have been observed to complete the intercellular communication in this way while preventing endogenous degradation (Turchinovich et al., 2011; Celluzzi and Masotti, 2016; Yu et al., 2016; Bartel, 2018; Moloney et al., 2019). miRNAs dysregulation observed in tissues and serum associated with disease activity was considered as a potential biomarker for clinical diagnosis (Link et al., 2010; Kalla et al., 2015; Rupaimoole and Slack, 2017; Tili et al., 2017; Hossian et al., 2019; Chen et al., 2020), but several issues have impeded the application of circulating miRNAs in clinical diagnosis (Wang et al., 2018; Saliminejad et al., 2019). In the host’s gastrointestinal tract, fecal miRNA can be influenced by the alteration of gut microbial composition, which provides a new perspective to identify intestinal and microbiota healthiness (Carter et al., 2017; Horne et al., 2019; Tarallo et al., 2019; Sarshar et al., 2020).

## The Implications of miRNA for the Host

MicroRNAs play an important role in the host’s gastrointestinal function. Numerous studies have revealed that miRNAs can participate in many aspects of intestinal functions, including proliferation and differentiation of cells, architecture, and barrier function of the host intestines (Zou et al., 2018; Ding et al., 2019). In 2010, McKenna et al. (2010) determined the complete miRNA transcriptome of the mouse intestinal epithelium cells. They found that miR-192 and let-7 presented high expression levels and observed reduction in goblet cells, increased apoptosis, and intestinal inflammation in the mice deficient of all miRNAs in the intestine. Part of the reason for the proliferation and differentiation of IECs is the precise regulation of key transcription factors, such as SOX9 (Peck et al., 2016). miR-30 could indirectly regulate SOX9 protein and thus mediate IEC homeostasis (Suez et al., 2019). Recent study showed that miR-381-3p directly suppressed nuclear receptor-related protein 1 (nurr1) translation (Liu et al., 2018). By inhibiting miR-381-3p, nurr1-mediated IEC proliferation and barrier function were enhanced.

With the increasing recognition of the importance of miRNAs in the host’s gastrointestinal tract function, the association between miRNAs and a wide variety of intestinal diseases have received more and more attention (Tables 1, 2). For example, colorectal cancer (CRC) is one of the most common malignant tumors. Multiple signaling pathways (WNT, EGFR, TGF-β, etc.) in CRC are regulated by miRNAs (Balacescu et al., 2018). They have been identified as oncomiRs and tumor suppressor miRNAs (Yamamoto and Mori, 2016; Balacescu et al., 2018; Shirafkan et al., 2018). Their deregulation contributes to the development, progression, and metastasis of CRC (Yamamoto and Mori, 2016; Balacescu et al., 2018; Shirafkan et al., 2018; To et al., 2018). Natural endogenous and stability make numerous of them therapeutic targets and potential biomarkers for CRC (Yamamoto and Mori, 2016; Shirafkan et al., 2018; To et al., 2018). The exploitation of favorable miRNAs delivery systems is likely to expand the options for CRC treatment. Similarly, many miRNAs could interfere in the pathogenesis of IBDs by regulating multiple pathways (Cao et al., 2017; Schönauen et al., 2018; Boros and Nagy, 2019; Feng et al., 2019). They were observed to play important roles in inflammation, fibrosis, autophagy, and cellular proliferation (Yamamoto and Mori, 2016; Balacescu et al., 2018; Shirafkan et al., 2018; To et al., 2018; Feng et al., 2019). According to Tian et al. (2019), miRNA 31 (miR31) expression was altered in inflamed mucosa of IBD patients *via* the NF-kB and STAT3 signaling pathways. This miRNA has the function of relieving inflammation and promoting epithelial cell regeneration. A microsphere delivery system has been developed based on this characteristic of miR31, which provided a new proposal for the treatment of IBD.

**TABLE 1:** Circulating miRNAs and their possible/identified targets in different gastrointestinal disorders.

**Circulating miRNAs**	**Diseases**	**Up/down**	**Potential/identified target genes**	**Roles**	**References**
miR-25-3p	CRC	Up	KLF2, KLF4	Promoting CRC vascular permeability and angiogenesis	Zeng et al., 2018
miR-106b-3p	CRC	Up	DLC-1	Promoting CRC metastasis	Liu et al., 2020
miR-1229	CRC	Up	HIPK2	Promoting CRC angiogenesis	Hu et al., 2019
miR-590-5p	CRC	Down	NF90	Inhibiting CRC angiogenesis and metastasis	Zhou Q. et al., 2016
miR-622	CRC	Down	CXCR4, K-Ras	Inhibiting CRC angiogenesis and metastasis	Fang et al., 2016, 2019
miR-382	CRC	Down	NR2F2	Inhibiting CRC cell growth and invasion	Zhou B. et al., 2016
miR-409-3p	CRC	Down	Beclin-1	Inhibiting autophagy	Tan et al., 2016
miR-223	IBD	Up	CLDN8	Dysfunction of the intestinal epithelial barrier	Wang et al., 2016
miR-21	IBD	Up	RhoB	Dysfunction of the intestinal epithelial barrier	Yang et al., 2013
miR-320	IBD	Down	NOD2	Disruption of immune homeostasis	Pierdomenico et al., 2016
miR-31	IBD	Up	IL6st, IL7r, IL17ra	Suppressing inflammation	Tian et al., 2019

*Up and down indicate upregulated and downregulated circulating miRNA expression levels in diseased tissues compared with normal tissues, respectively. Roles, roles of miRNAs in the pathogenesis of gastrointestinal disorders; CRC, colorectal cancer; IBD, inflammatory bowel disease.*

**TABLE 2:** Fecal miRNAs and bacteria correlated with them in different gastrointestinal disorders.

**Fecal miRNAs**	**Diseases**	**Up/down**	**Correlated microbiome taxa**	**References**
miRNA-135b	CRC	Up	–	Wu et al., 2014
miR-21	CRC	Up	–	Link et al., 2010
miR-106a	CRC	Up	–	Link et al., 2010
miR-182	CRC	Up	*Blautia*	Yuan et al., 2018
miR-139	CRC	Down	*Blautia*	Yuan et al., 2018
miR-155	IBD	Up	–	Schönauen et al., 2018
miR-223	IBD	Up	–	Schönauen et al., 2018
miR-199a	IBD	Up	–	Ji et al., 2018
miR-223-3p	IBD	Up	–	Ji et al., 2018
miR-1226	IBD	Down	–	Ji et al., 2018
miR-515-5p	IBD	Down	–	Ji et al., 2018

*Up and down indicate upregulated and downregulated fecal miRNA expression levels in diseased tissues compared with normal tissues, respectively. Correlated microbiome taxa, bacteria significantly correlated with fecal miRNAs; CRC, colorectal cancer; IBD, inflammatory bowel disease.*

Notably, microbiota-derived extracellular vesicles (EVs) also play a major role in intercellular communication and signal transduction between gut microbiota and host cells. EVs included outer membrane vesicles (OMVs) and membrane vesicles (MVs) that are, respectively, liberated by Gram-negative and Gram-positive bacteria. Various RNA species (e.g., mRNAs, miRNAs, tRNAs) are biologically active components of EVs, which may impact gene expression when being delivered to host cells (Ahmadi Badi et al., 2017; Felli et al., 2017; Macia et al., 2019; Sarshar et al., 2020). In a fascinating study, Fábrega et al. confirmed the role of OMVs in signal transduction between gut microbiota and host. OMVs produced by the probiotic *Escherichia coli* strain Nissle (EcN) 1917 induced the expression and secretion of several cytokines and chemokines in the *ex vivo* model (Fábrega et al., 2016). Overall, intestinal bacteria utilized OMVs as a significant strategy to communicate with the host and influence host responses.

## The Correlation Between Host miRNA Expression Levels and Gut Microbiota Profiles

In addition to participating in the regulation of intestinal function, the close relationship between miRNAs and gut microbiota has also been confirmed (Masotti, 2012; Belcheva, 2017; Hoban et al., 2017; Yuan et al., 2018). A potential mechanism by which gut microbiota affect host physiology is to modulate host gene expression through miRNAs. Dalmasso et al. (2011) demonstrated that nine miRNAs were differently expressed in the colonized mice relative to germ-free mice. They confirmed that the potential targets of miRNAs overlapped with the dysregulated host genes during microbial colonization. Commensal bacteria might affect more host genes at the post-transcriptional level than expected. Singh et al. (2012) investigated the influence of endogenous microbiota on the overall expression of cecal miRNA *in vivo* by using germ-free and conventional mice. The result supported that cecal miRNA expression levels were modified by endogenous microbiota. The authors also discovered the proteins encoded by 34 putative miRNA targets involved in controlling intestinal barrier and immune regulation. Also, Xue et al. (2011) reported a study focusing on the roles of commensal bacteria in the regulation of intestinal gene expression. They found that commensal bacteria downregulated dendritic cell miR-10a expression *via* TLR–TLR ligand interactions through a MyD88-dependent pathway. IL-12/IL-23p40, a key molecule for innate immune responses to commensal bacteria, was identified as a target gene for miR-10a. Compared with the control mice, colitis mice have higher levels of IL-12/IL-23p40 and lower levels of intestinal miR-10a. Surprisingly, gut microbiota are also reported to influence host miRNA expression in other tissues and organs (Zhou G. et al., 2017; Allegra et al., 2020). Both the two studies have identified the effects of gut microbiota on the miRNA expression in the hippocampus and white adipocytes in mice, respectively (Chen J.J. et al., 2017; Virtue et al., 2019). An additional researcher claimed that after viral infection, the expression of miRNAs in the lungs of antibiotic-treated mice was altered, resulting in a reduction in host antiviral immunity (Pang et al., 2018). Accordingly, the aforementioned findings indicated that miRNA could impact the host through participating in signal transmission, maintaining intestinal homeostasis.

## Interactions Between Host miRNAs and Bacterial Pathogens

miRNAs have also been recognized for their important role in the interactions between host and bacterial pathogens, either as an indispensable part of the host response to fight infection or as a molecular strategy utilized by bacterial pathogens to cause the dysregulation of host miRNA expression for their own benefit (Aguilar et al., 2019a,b). For example, miR-301b is involved in the augmentation of pro-inflammatory response during infection by *Pseudomonas aeruginosa* (Li X. et al., 2016). A target of miR-301b is c-Myb that can increase the expression of anti-inflammatory cytokines IL-4 and TGF-β1. miR-301b suppresses anti-inflammatory response by targeting c-Myb in response to *P. aeruginosa* infection. Wang et al. (2017) observed that miR-143-3p expression was significantly upregulated in *Helicobacter pylori*-positive gastric cancer tissues. miR-143-3p targets AKT2, which induced cell apoptosis and negatively regulated tumor growth, migration, and invasion. Bacterial pathogens have evolved the ability to regulate host miRNAs to resist autophagy, thereby promoting the survival and reproduction of bacteria. miR-23a-5p was shown to facilitate bacterial survival and inhibit *Mycobacterium tuberculosis*-induced autophagy in macrophages by impacting the TLR2/MyD88/NF-κB pathway (Gu et al., 2017). miRNAs hav emerged as important participants in the interactions between host and bacterial pathogens, which suggests an underlying mechanism that miRNAs are transmitted between host and bacteria.

## Fecal miRNA and Plant-Derived miRNA as Useful Tools for Maintaining Gut Homeostasis

Actually, a bidirectional relationship exists between gut microbiome and miRNAs. An analysis of miRNA sequencing in human CRC tumor and normal tissues identified 76 differentially expressed miRNAs that are correlated with the abundance of microbes in the tumor microenvironment, including Firmicutes, Bacteroidetes, and Proteobacteria (Yuan et al., 2018). Moloney et al. (2018) proved that the expression of murine miRNAs produced by IECs in feces was influenced by the gut microbiota (Gu et al., 2017). They discovered that the relative abundance of the phyla Bacteroidetes and Firmicutes was significantly correlated with the level of miR-141-3p and phyla Actinobacteria, Bacteroidetes, Cyanobacteria, Firmicutes, and Proteobacteria were significantly correlated with the level of miR-200a-3p. As a valuable tool, host miRNAs play significant roles in maintaining intestinal homeostasis (Link et al., 2010; Moloney et al., 2018; Yuan et al., 2018). Recent studies have illustrated the mechanism by which the host could foster favorable microbiota *via* fecal miRNA (Liu et al., 2016; Jiayi et al., 2019). An important work from Liu et al. introduced that fecal miRNAs are mainly derived from IECs and Hopx+ cells (Liu et al., 2016). miRNAs present in the feces have the ability to enter bacterial cells and specifically regulate gene transcripts, thereby affecting the growth of bacteria. miRNA mimics synthesized for oral administration could act on gut bacteria. Moreover, they found that IEC miRNA-deficient mice suffered from dysbiosis and that the fecal miRNA transplantation from wild-type mice restored the intestinal bacterial community. Recently, Liu and Weiner (2016) also reported that fecal miR-30d upregulated the expression of a lactase in *Akkermansia muciniphila* and increased this bacterial abundance in the gut, which suppressed multiple sclerosis in mouse model through the expansion of regulatory T cells (Liu and Weiner, 2016).

Dietary intervention is widely known as an important means for the host to maintain intestinal homeostasis (Liu et al., 2019). Another source of miRNAs that the host can utilize is plant-derived miRNAs. These miRNAs from food can also enter bacteria and regulate gene expression. Teng et al. revealed that miRNAs encapsulated in plant-derived exosome-like nanoparticles (ELNs) were taken up by gut bacteria, subsequently bound to bacterial mRNAs, and modified the composition of gut microbiota (Nagai et al., 2016). The priority for specific gut bacteria to absorb plant-derived ELNs depended on the lipid type of the outer membrane of ELNs. More importantly, miRNAs within ginger ELNs could enter *Lactobacillus rhamnosus* GG (LGG) and induce the IL-22 expression *via* the activation of the AHR pathway to enhance LGG-mediated inhibition of mouse colitis (Spinler et al., 2019). This result indicated that probiotics may be manipulated by miRNA originating from the host or diet.

In summary, these data demonstrated that the fecal miRNA and plant-derived miRNA contribute to the modification of the gut microbiome.

## Exploration of miRNA-Based Molecular Mechanism of Probiotics Action

Host–microbiota interactions play a vital role in intestinal homeostasis, and miRNAs have been considered to be key molecular regulators for mediating such interactions (Behrouzi et al., 2020). The addition of probiotics can also interfere with these interactions and influence the expression of miRNAs (Teng et al., 2018). For instance, miRNAs involved in the alleviation of cecal inflammation are induced by probiotic *Lactobacillus plantarum* Z01 (Rodríguez-Nogales et al., 2018a). In IBD, probiotic EcN 1917 was reported to have the ability to regulate the miRNA expression levels that participated in the inflammatory response in colitic mice (Chen Q. et al., 2017). Similarly, *Bacillus coagulans* R11 may alter the structure of the bacterial community by influencing the host fecal miRNAs (Rodríguez-Nogales et al., 2018b). The ability to regulate miRNA expression by probiotics is of great significance for maintaining the intestinal microenvironment homeostasis. Notably, there is a highly complex relationship between miRNAs and intestinal diseases. However, few reports are available about the roles of miRNAs in shaping the gut microbiota as a therapy of diseases. Moreover, the capacity of the host to regulate gut microbiota in a miRNA-dependent manner remains poorly understood and may even be restricted. Considering that a health benefit was delivered to the host *via* the ingestion of probiotics, the effects of miRNAs may be strengthened by these beneficial microorganisms.

**FIGURE 3:**
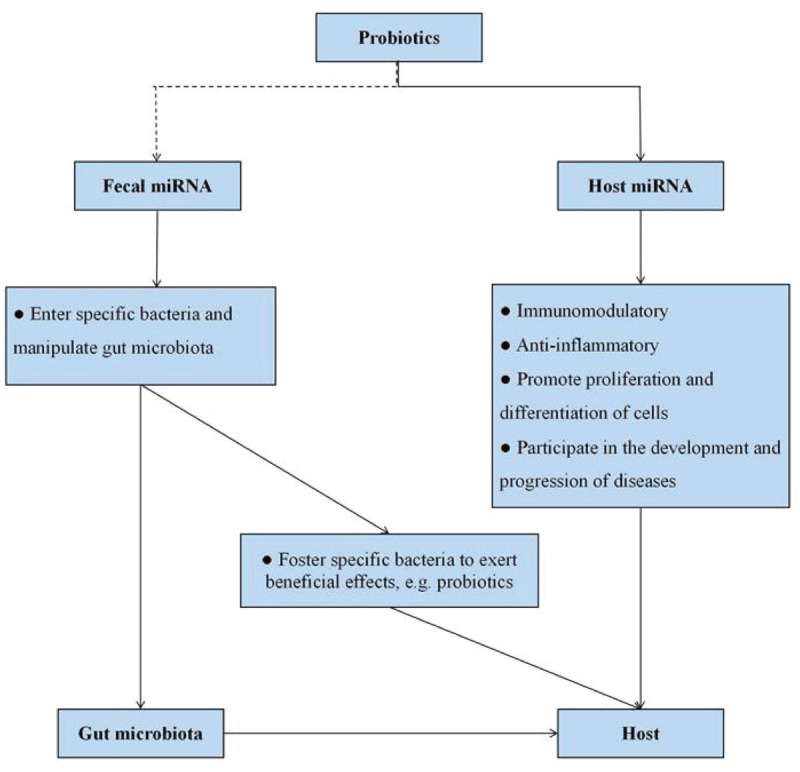
Potential miRNA-based molecular mechanism of probiotics action. Probiotics can influence host miRNA expression, thereby favoring numerous host functions. On the other hand, host cells foster specific bacteria to control intestinal homeostasis and exert beneficial effects on the host itself *via* fecal miRNA and plant-derived miRNA. The dotted line represents the lack of conclusive evidence in the relationship at present.

As mentioned above, miRNAs have emerged as important mediators of interactions between host and gut microbiota. Furthermore, probiotics have the ability to modulate miRNA expression levels, and we speculated that the potential mechanism of probiotics acting on the host may be associated with the modification of miRNAs (Figures 1, 3). Probiotics drive intestinal cells to produce miRNAs with important regulatory functions (such as intestinal anti-inflammatory effects) to act on host cells. On the other hand, probiotics impact the releases of fecal miRNAs from IECs, thereby prompting fecal miRNAs to penetrate into specific bacteria and regulate gene expression, with consequences for restoring the “signature” of gut microbiota.

Additionally, side effects of probiotics should not be ignored. Although probiotics are widely used and have considerable potential as preventive or therapeutic options against various gastrointestinal disorders, there are still a few reports indicating the potentially negative effects of probiotics. In fact, the reason for these conflicting clinical results is that the health benefits provided by probiotics to the host depend on many factors, including the strains, individuals, types of administration, or location within the intestines. At present, there is no sufficient and convincing evidence to demonstrate the mechanism of probiotics resulting in negative consequences in the host. The exact mechanisms of both positive and negative effects of probiotics should be further investigated and perfected. Here, this review aimed to open up a new direction for studying the molecular pathways involved in the probiotic mode of action. More explorations and evidence for the research about elucidating the probiotic mechanisms of action are desirable.

## Future Perspectives

In recent years, significant breakthroughs have been made in miRNA-based research field, especially in regard to the applications that provided opportunities for the treatment of numerous gastrointestinal diseases including CRC, IBD, etc. (Soroosh et al., 2018; Konno et al., 2019; Tian et al., 2019; Xing et al., 2019). Notably, a single miRNA can target multiple mRNAs, and one mRNA can become the target of numerous miRNAs. A wide range of miRNAs from mammalian cells constitute a complex regulatory network. In this context, it is particularly important to clarify the real targets with therapeutic effects. They will be valuable tools for the treatment and intervention of patients with various diseases. miRNA-based therapeutics are able to deliver miRNA mimics or anti-miRs to target tissues and target cells through advanced technologies. Several pathways have shown promise to enable this strategy to become a clinical reality. Simultaneously, the administration of fecal miRNA mimics or anti-miRs *in vivo* makes manipulation of the gut microbiota feasible. However, this concept is still relatively novel in the field, and the investigation of manipulating bacterial communities has just begun. Growing valuable insights will promote the cross-kingdom communication between host and gut microorganism to become a subject of intense exploration.

To date, accumulating evidence supported that miRNAs play central roles in mediating host–microbe interactions and can be used as biomarkers of intestinal and microbiota healthiness. Considering that probiotics are likely to be the regulators or amplifiers of miRNA expression levels, we believe that miRNA may be an important part of the molecular mechanism underlying how probiotics maintain gut homeostasis. In this point, the probiotics–miRNA–host/gut microbiota axis can serve as a new direction for the next phase of research but with the caveat that this molecular mechanism of probiotics remains to be fully understood. Further work is needed to delineate the precise mechanism of how probiotics control miRNA expression levels and to obtain more knowledge about subsequently how these prominently expressed miRNAs mediate host–microbe interactions.

## Author Contributions

YZh, YZe, and XN contributed to the topic conception. YZh wrote the main manuscript text. YZe designed the figures. YZh and MZ revised the manuscript. All authors read and approved the final manuscript.

## Conflict of Interest

The authors declare that the research was conducted in the absence of any commercial or financial relationships that could be construed as a potential conflict of interest.

## References

[B1] Abid M. B. Koh C. J. (2019). Probiotics in health and disease: fooling Mother Nature? *Infection* 47 911–917. 10.1007/s15010-019-01351-0 31478123

[B2] Adak A. Khan M. R. (2019). An insight into gut microbiota and its functionalities. *Cell. Mol. Life Sci.* 76 473–493. 10.1007/s00018-018-2943-4 30317530 PMC11105460

[B3] Aguilar C. Mano M. Eulalio A. (2019a). MicroRNAs at the host–bacteria interface: host defense or bacterial offense. *Trends Microbiol.* 27 206–218. 10.1016/j.tim.2018.10.011 30477908

[B4] Aguilar C. Mano M. Eulalio A. (2019b). Multifaceted roles of microRNAs in host-bacterial pathogen interaction. *Microbiol. Spectr.* 7:31152522. 10.1128/microbiolspec.bai-0002-2019 PMC11026079 31152522

[B5] Agus A. Planchais J. Sokol H. (2018). Gut microbiota regulation of tryptophan metabolism in health and disease. *Cell Host. Microbe.* 23 716–724. 10.1016/j.chom.2018.05.003 29902437

[B6] Ahmadi Badi S. Moshiri A. Fateh A. Rahimi Jamnani F. Sarshar M. Vaziri F. (2017). Microbiota-derived extracellular vesicles as new systemic regulators. *Front. Microbiol.* 8:1610. 10.3389/fmicb.2017.01610 28883815 PMC5573799

[B7] Allaire J. M. Crowley S. M. Law H. T. Chang S. Y. Ko H. J. Vallance B. A. (2018). The intestinal epithelium: central coordinator of mucosal immunity. *Trends Immunol.* 39 677–696. 10.1016/j.it.2018.04.002 29716793

[B8] Allegra A. Musolino C. Tonacci A. Pioggia G. Gangemi S. (2020). Interactions between the microRNAs and microbiota in cancer development: roles and therapeutic opportunities. *Cancers* 12:805 10.3390/cancers12040805 PMC7225936 32230762

[B9] Balacescu O. Sur D. Cainap C. Visan S. Cruceriu D. Manzat-Saplacan R. (2018). The impact of miRNA in colorectal cancer progression and its liver metastases. *Int. J. Mol. Sci.* 19:3711. 10.3390/ijms19123711 30469518 PMC6321452

[B10] Balakrishnan M. Floch M. H. (2012). Prebiotics, probiotics and digestive health. *Curr. Opin. Clin. Nutr. Metab. Care* 5 580–585. 10.1097/MCO.0b013e328359684f 23037903

[B11] Bartel D. P. (2018). Metazoan microRNAs. *Cell* 173 20–51. 10.1016/j.cell.2018.03.006 29570994 PMC6091663

[B12] Behrouzi A. Ashrafian F. Mazaheri H. Lari A. Nouri M. Riazi Rad F. (2020). The importance of interaction between microRNAs and gut microbiota in several pathways. *Microb. Pathog.* 144:104200 10.1016/j.micpath.2020.104200 32289465

[B13] Belcheva A. (2017). MicroRNAs at the epicenter of intestinal homeostasis. *Bioessays* 39:28155997. 10.1002/bies.201600200 28155997

[B14] Bermudez-Brito M. Plaza-Díaz J. Muñoz-Quezada S. Gómez-Llorente C. Gil A. (2019). Probiotic mechanisms of action. *Early Hum. Dev.* 135 58–65. 10.1016/j.earlhumdev.2019.05.010 31174927

[B15] Boros É Nagy I. (2019). The role of microRNAs upon epithelial-to-mesenchymal transition in inflammatory bowel disease. *Cells* 8:1461. 10.3390/cells8111461 31752264 PMC6912477

[B16] Brown E. M. Manish S. Brett F. (2013). The role of the immune system in governing host-microbe interactions in the intestine. *Nat. Immunol.* 14 660–667. 10.1038/ni.2611 23778793

[B17] Cao B. Zhou X. Ma J. Zhou W. Yang W. Fan D. (2017). Role of miRNAs in inflammatory bowel disease. *Dig. Dis. Sci.* 62 1426–1438. 28391412 10.1007/s10620-017-4567-1

[B18] Carter J. V. Galbraith N. J. Yang D. Burton J. F. Walker S. P. Galandiuk S. (2017). Blood-based microRNAs as biomarkers for the diagnosis of colorectal cancer: a systematic review and meta-analysis. *Br. J. Cancer* 116 762–774. 10.1038/bjc.2017.12 28152545 PMC5355921

[B19] Celiker C. Kalkan R. (2020). Genetic and epigenetic perspective of microbiota. *Appl. Microbiol. Biotechnol.* 104 8221–8229. 10.1007/s00253-020-10849-9 32857199

[B20] Celluzzi A. Masotti A. (2016). How our other genome controls our epi-genome. *Trends Microbiol.* 24 777–787. 10.1016/j.tim.2016.05.005 27289569

[B21] Chang C. S. Kao C. Y. (2019). Current understanding of the gut microbiota shaping mechanisms. *J. Biomed. Sci.* 26:59. 10.1186/s12929-019-0554-5 PMC6702754 31434568

[B22] Chen J. J. Zeng B. H. Li W. W. Zhou C. J. Fan S. H. Cheng K. (2017). Effects of gut microbiota on the microRNA and mRNA expression in the hippocampus of mice. *Behav. Brain Res.* 322 34–41. 10.1016/j.bbr.2017.01.021 28093256

[B23] Chen P. Zhou G. Lin J. Li L. Zeng Z. Chen M. (2020). Serum biomarkers for inflammatory bowel disease. *Front. Med.* 7:123 10.3389/fmed.2020.00123 PMC7188783 32391365

[B24] Chen Q. Tong C. Ma S. Zhou L. Zhao L. Zhao X. (2017). Involvement of microRNAs in probiotics-induced reduction of the cecal inflammation by *Salmonella Typhimurium*. *Front. Immunol.* 8:704. 10.3389/fimmu.2017.00704 28659929 PMC5468434

[B25] Chu H. Mazmanian S. K. (2013). Innate immune recognition of the microbiota promotes host-microbial symbiosis. *Nat. Immunol.* 14 668–675. 10.1038/ni.2635 23778794 PMC4109969

[B26] Coyte K. Z. Schluter J. Foster K. R. (2015). The ecology of the microbiome: networks, competition, and stability. *Science* 350 663–666. 10.1126/science.aad2602 26542567

[B27] Cremon C. Barbaro M. R. Ventura M. Barbara G. (2018). Pre- and probiotic overview. *Curr. Opin. Pharmacol.* 43 87–92. 10.1016/j.coph.2018.08.010 30219638

[B28] Cristofori F. Indrio F. Miniello V. L. De Angelis M. Francavilla R. (2018). Probiotics in celiac disease. *Nutrients* 10:1824. 10.3390/nu10121824 30477107 PMC6316269

[B29] Cuevas-Sierra A. Ramos-Lopez O. Riezu-Boj J. I. Milagro F. I. Martinez J. A. (2019). Diet, gut microbiota, and obesity: links with host genetics and epigenetics and potential applications. *Adv. Nutr.* 10 S17–S30. 30721960 10.1093/advances/nmy078 PMC6363528

[B30] Dalmasso G. Nguyen H. T. Yan Y. Laroui H. Charania M. A. Ayyadurai S. (2011). Microbiota modulate host gene expression via microRNAs. *PLoS One.* 6:e19293. 10.1371/journal.pone.0019293 21559394 PMC3084815

[B31] Ding S. Liu G. Jiang H. Fang J. (2019). MicroRNA determines the fate of intestinal epithelial cell differentiation and regulates intestinal diseases. *Curr. Protein. Pept. Sci.* 20 666–673. 10.2174/1389203720666190125110626 30678626

[B32] Dobson A. Cotter P. D. Ross R. P. Hill C. (2012). Bacteriocin production: a probiotic trait? *Appl. Environ. Microbiol.* 78 1–6. 10.1128/aem.05576-11 22038602 PMC3255625

[B33] Du L. Liu F. Ju X. Huo G. (2010). Adhesion capability of first two domains at N terminus of NP_785232 protein and their interaction with a UV-absorbing component from human mucus. *Lett. Appl. Microbiol.* 51 400–405. 10.1111/j.1472-765x.2010.02911.x 20840550

[B34] El Kaoutari A. Armougom F. Gordon J. I. Raoult D. Henrissat B. (2013). The abundance and variety of carbohydrate-active enzymes in the human gut microbiota. *Nat. Rev. Microbiol.* 11 497–504. 10.1038/nrmicro3050 23748339

[B35] Fábrega M. J. Aguilera L. Giménez R. Varela E. Alexandra Cañas M. Antolín M. (2016). Activation of immune and defense responses in the intestinal mucosa by outer membrane vesicles of commensal and probiotic *Escherichia coli* Strains. *Front. Microbiol.* 7:705. 10.3389/fmicb.2016.00705 27242727 PMC4863414

[B36] Fang Y. Sun B. Li Z. Chen Z. Xiang J. (2016). MiR-622 inhibited colorectal cancer occurrence and metastasis by suppressing K-Ras. *Mol. Carcinog.* 55 1369–1377. 10.1002/mc.22380 26333174

[B37] Fang Y. Sun B. Wang J. Wang Y. (2019). miR-622 inhibits angiogenesis by suppressing the CXCR4-VEGFA axis in colorectal cancer. *Gene* 699 37–42. 10.1016/j.gene.2019.03.004 30851425

[B38] Felli C. Baldassarre A. Masotti A. (2017). Intestinal and circulating microRNAs in coeliac disease. *Int. J. Mol. Sci.* 18:1907. 10.3390/ijms18091907 28878141 PMC5618556

[B39] Feng Y. Zhang Y. Zhou D. Chen G. Li N. (2019). MicroRNAs, intestinal inflammatory and tumor. *Bioorg. Med. Chem. Lett.* 29 2051–2058. 10.1016/j.bmcl.2019.06.013 31213403

[B40] Fischer S. E. J. (2015). RNA interference and microRNA-mediated silencing. *Curr. Protoc. Mol. Biol.* 112 26.1.1–26.1.5. 26423588 10.1002/0471142727.mb2601s112

[B41] Forgie A. J. Fouhse J. M. Willing B. P. (2019). Diet-microbe-host interactions that affect gut mucosal integrity and infection resistance. *Front. Immunol.* 10:1802. 10.3389/fimmu.2019.01802 31447837 PMC6691341

[B42] Gentile C. L. Weir T. L. (2018). The gut microbiota at the intersection of diet and human health. *Science* 362 776–780. 10.1126/science.aau5812 30442802 PMC13264711

[B43] Gómez-Llorente C. Muñoz S. Gil A. (2010). Role of Toll-like receptors in the development of immunotolerance mediated by probiotics. *Proc. Nutr. Soc.* 69 381–389. 10.1017/s0029665110001527 20416121

[B44] Gresse R. Chaucheyras-Durand F. Fleury M. A. Van de Wiele T. Forano E. Blanquet-Diot S. (2017). Gut microbiota dysbiosis in postweaning piglets: understanding the keys to health. *Trends Microbiol.* 25 851–873. 10.1016/j.tim.2017.05.004 28602521

[B45] Gu X. Gao Y. Mu D. G. Fu E. Q. (2017). MiR-23a-5p modulates mycobacterial survival and autophagy during *mycobacterium tuberculosis* infection through TLR2/MyD88/NF-κB pathway by targeting TLR2. *Exp. Cell Res.* 354 71–77. 10.1016/j.yexcr.2017.03.039 28327409

[B46] Gura T. (2014). Nature’s first functional food. *Science* 345 747–749. 10.1126/science.345.6198.747 25124424

[B47] Hasan N. Yang H. (2019). Factors affecting the composition of the gut microbiota, and its modulation. *PeerJ.* 7:e7502. 10.7717/peerj.7502 31440436 PMC6699480

[B48] Hill C. Guarner F. Reid G. Gibson G. R. Merenstein D. J. Pot B. (2014). Expert consensus document. Te International Scientifc Association for Probiotics and Prebiotics consensus statement on the scope and appropriate use of the term probiotic. *Nat. Rev. Gastroenterol. Hepatol.* 11 506–514. 10.1038/nrgastro.2014.66 24912386

[B49] Hoban A. E. Stilling R. M. Moloney G. M. Moloney R. D. Shanahan F. (2017). Microbial regulation of microRNA expression in the amygdala and prefrontal cortex. *Microbiome* 5:102. 10.1186/s40168-017-0321-3 PMC5571609 28838324

[B50] Horne R. St Pierre J. Odeh S. Surette M. Foster J. A. (2019). Microbe and host interaction in gastrointestinal homeostasis. *Psychopharmacology* 236 1623–1640. 10.1007/s00213-019-05218-y 30900006 PMC6599184

[B51] Hossian A. K. M. N. Mackenzie G. G. Mattheolabakis G. (2019). miRNAs in gastrointestinal diseases: can we effectively deliver RNA-based therapeutics orally? *Nanomedicine* 14 2873–2889. 10.2217/nnm-2019-0180 31735124 PMC7026766

[B52] Hu H. Y. Chen-Huan Y. Huan-Huan Z. Song-Zhao Z. Wen-Ying Y. (2019). Exosomal miR-1229 derived from colorectal cancer cells promotes angiogenesis by targeting HIPK2. *Int. J. Biol. Macromol.* 132 470–477. 10.1016/j.ijbiomac.2019.03.221 30936013

[B53] Ji Y. Li X. Zhu Y. Li N. Zhang N. Niu M. (2018). Faecal microRNA as a biomarker of the activity and prognosis of inflammatory bowel diseases. *Biochem. Biophys. Res. Commun.* 503 2443–2450. 10.1016/j.bbrc.2018.06.174 29969632

[B54] Jiayi D. Jesse W. T. Li-Fan L. (2019). miRNA-microbiota interaction in gut homeostasis and colorectal cancer. *Trends Cancer* 5 666–669. 10.1016/j.trecan.2019.08.003 31735285 PMC8480531

[B55] Kalla R. Ventham N. T. Kennedy N. A. Quintana J. F. Nimmo E. R. Buck A. H. (2015). MicroRNAs: new players in IBD. *Gut* 64 504–517. 10.1136/gutjnl-2014-307891 25475103 PMC4345829

[B56] Khoruts A. (2018). Targeting the microbiome: from probiotics to fecal microbiota transplantation. *Genome Med.* 10:80. 10.1186/s13073-018-0592-8 PMC6208019 30376869

[B57] Kiousi D. E. Karapetsas A. Karolidou K. Panayiotidis M. I. Pappa A. Galanis A. (2019). Probiotics in extraintestinal diseases: current trends and new directions. *Nutrients* 11:788. 10.3390/nu11040788 30959761 PMC6521300

[B58] Konno M. Koseki J. Asai A. Yamagata A. Shimamura T. Motooka D. (2019). Distinct methylation levels of mature microRNAs in gastrointestinal cancers. *Nat. Commun.* 10:3888. 10.1038/s41467-019-11826-1 PMC6715669 31467274

[B59] Kothari D. Patel S. Kim S. K. (2019). Probiotic supplements might not be universally-effective and safe: A review. *Biomed. Pharmacother.* 111 537–547. 10.1016/j.biopha.2018.12.104 30597307

[B60] Langella P. Chatel J. M. (2019). Risk assessment of probiotics use requires clinical parameters. *Nat. Rev. Gastroenterol. Hepatol.* 16 202–204. 10.1038/s41575-019-0111-4 30692658

[B61] LeBlanc J. G. Chain F. Martín R. Bermúdez-Humarán L. G. Courau S. Langella P. (2017). Beneficial effects on host energy metabolism of short-chain fatty acids and vitamins produced by commensal and probiotic bacteria. *Microb. Cell Fact.* 16:79. 10.1186/s12934-017-0691-z PMC5423028 28482838

[B62] Lee E. S. Song E. J. Nam Y. D. Lee S. Y. (2018). Probiotics in human health and disease: from nutribiotics to pharmabiotics. *J. Microbiol.* 56 773–782. 10.1007/s12275-018-8293-y 30353462

[B63] Lee H. (2019). Microbe-host communication by small RNAs in extracellular vesicles: vehicles for transkingdom RNA transportation. *Int. J. Mol. Sci.* 20:1487. 10.3390/ijms20061487 30934547 PMC6472211

[B64] Li D. Wang P. Wang P. Hu X. Chen F. (2016). The gut microbiota: A treasure for human health. *Biotechnol. Adv.* 34 1210–1224. 10.1016/j.biotechadv.2016.08.003 27592384

[B65] Li X. He S. Li R. Zhou X. Zhang S. Yu M. (2016). *Pseudomonas aeruginosa* infection augments inflammation through miR-301b repression of c-Myb-mediated immune activation and infiltration. *Nat. Microbiol.* 1:16132. 10.1038/nmicrobiol.2016.132 PMC5061341 27670114

[B66] Link A. Balaguer F. Shen Y. Nagasaka T. Lozano J. J. Boland C. R. (2010). Fecal MicroRNAs as novel biomarkers for colon cancer screening. *Cancer Epidemiol. Biomar. Prev.* 19 1766–1774. 10.1158/1055-9965.epi-10-0027 20551304 PMC2901410

[B67] Liu H. Liu Y. Sun P. Leng K. Xu Y. Mei L. (2020). Colorectal cancer-derived exosomal miR-106b-3p promotes metastasis by down-regulating DLC-1 expression. *Clin. Sci.* 134 419–434. 10.1042/cs20191087 32065214

[B68] Liu L. Yao J. Li Z. Zu G. Feng D. Li Y. (2018). miR-381-3p knockdown improves intestinal epithelial proliferation and barrier function after intestinal ischemia/reperfusion injury by targeting nurr1. *Cell Death Dis.* 9:411. 10.1038/s41419-018-0450-z PMC5852084 29540663

[B69] Liu S. (2016). The development of our organ of other kinds-the gut microbiota. *Front. Microbiol.* 7:2107. 10.3389/fmicb.2016.02107 28066404 PMC5179505

[B70] Liu S. Weiner H. L. (2016). Control of the gut microbiome by fecal microRNA. *Microb. Cell.* 3 176–177. 10.15698/mic2016.04.492 28357349 PMC5349091

[B71] Liu S. da Cunha A. P. Rezende R. M. Cialic R. Wei Z. Bry L. (2016). The host shapes the gut microbiota via fecal microRNA. *Cell Host. Microbe.* 19 32–43. 10.1016/j.chom.2015.12.005 26764595 PMC4847146

[B72] Liu S. Rezende R. M. Moreira T. G. Tankou S. K. Cox L. M. Wu M. (2019). Oral administration of miR-30d from feces of MS patients suppresses MS-like symptoms in mice by expanding *Akkermansia muciniphila*. *Cell Host. Microbe.* 26 779–794.e. 31784260 10.1016/j.chom.2019.10.008 PMC6948921

[B73] Liwinski T. Elinav E. (2020). Harnessing the microbiota for therapeutic purposes. *Am. J. Transplant.* 20 1482–1488. 10.1111/ajt.15753 31858698

[B74] Long S. L. Gahan C. G. M. Joyce S. A. (2017). Interactions between gut bacteria and bile in health and disease. *Mol. Aspects Med.* 56 54–65. 10.1016/j.mam.2017.06.002 28602676

[B75] Lozupone C. A. Stombaugh J. I. Gordon J. I. Jansson J. K. Knight R. (2012). Diversity, stability and resilience of the human gut microbiota. *Nature* 489 220–230. 10.1038/nature11550 22972295 PMC3577372

[B76] Lu T. X. Rothenberg M. E. (2018). MicroRNA. *J. Allergy Clin. Immunol.* 141 1202–1207. 29074454 10.1016/j.jaci.2017.08.034 PMC5889965

[B77] Macia L. Nanan R. Hosseini-Beheshti E. Grau G. E. (2019). Host-and microbiota-derived extracellular vesicles, immune function, and disease development. *Int. J. Mol. Sci.* 21:107. 10.3390/ijms21010107 31877909 PMC6982009

[B78] Markowiak P. Śliżewska K. (2017). Effects of probiotics, prebiotics, and synbiotics on human health. *Nutrients* 9:1021. 10.3390/nu9091021 28914794 PMC5622781

[B79] Maslowski K. M. (2019). Metabolism at the centre of the host–microbe relationship. *Clin. Exp. Immunol.* 197 193–204. 10.1111/cei.13329 31107965 PMC6642865

[B80] Masotti A. (2012). Interplays between gut microbiota and gene expression regulation by miRNAs. *Front. Cell Infect. Microbiol.* 2:137. 10.3389/fcimb.2012.00137 23130352 PMC3487149

[B81] Matsuki T. Pédron T. Regnault B. Mulet C. Hara T. Sansonetti P. J. (2013). Epithelial cell proliferation arrest induced by lactate and acetate from *Lactobacillus casei* and *Bifidobacterium breve*. *PLoS One.* 8:e63053. 10.1371/journal.pone.0063053 23646174 PMC3639975

[B82] McKenna L. B. Schug J. Vourekas A. McKenna J. B. Bramswig N. C. Friedman J. R. (2010). MicroRNAs control intestinal epithelial differentiation, architecture, and barrier function. *Gastroenterology* 139 1654–1664.e1. 20659473 10.1053/j.gastro.2010.07.040 PMC3156097

[B83] Milani C. Duranti S. Bottacini F. Casey E. Turroni F. Mahony J. (2017). The first microbial colonizers of the human gut: composition, activities, and health implications of the infant gut microbiota. *Microbiol. Mol. Biol. Rev.* 81 e36–e17. 10.1128/MMBR.00036-17 PMC5706746 29118049

[B84] Mohr A. M. Mott J. L. (2015). Overview of microRNA biology. *Semin. Liver Dis.* 35 3–11. 10.1007/978-3-540-78709-9_1 25632930 PMC4797991

[B85] Moloney G. M. Dinan T. G. Clarke G. Cryan J. F. (2019). Microbial regulation of microRNA expression in the brain–gut axis. *Curr. Opin. Pharmacol.* 48 120–126. 10.1016/j.coph.2019.08.005 31590111

[B86] Moloney G. M. Viola M. F. Hoban A. E. Dinan T. G. Cryan J. F. (2018). Faecal microRNAs: indicators of imbalance at the host-microbe interface? *Benef. Microbes.* 9 175–183. 10.3920/bm2017.0013 29264965

[B87] Nagai M. Obata Y. Takahashi D. Hase K. (2016). Fine-tuning of the mucosal barrier and metabolic systems using the diet-microbial metabolite axis. *Int. Immunopharmacol.* 37 79–86. 10.1016/j.intimp.2016.04.001 27133028

[B88] Pang P. Yu B. Shi Y. Deng L. Xu H. Wu S. (2018). Alteration of intestinal flora stimulates pulmonary microRNAs to interfere with host antiviral immunity in influenza. *Molecules* 23:3151. 10.3390/molecules23123151 30513647 PMC6321108

[B89] Parasar B. Zhou H. Xiao X. Shi Q. Brito I. L. Chang P. V. (2019). Chemoproteomic profiling of gut microbiota-associated bile salt hydrolase activity. *ACS Cent Sci.* 5 867–873. 31139722 10.1021/acscentsci.9b00147 PMC6535767

[B90] Parker A. Lawson M. A. E. Vaux L. Pin C. (2018). Host-microbe interaction in the gastrointestinal tract. *Environ. Microbiol.* 20 2337–2353. 10.1111/1462-2920.13926 28892253 PMC6175405

[B91] Peck B. C. Sincavage J. Feinstein S. Mah A. T. Simmons J. G. Lund P. K. (2016). miR-30 family controls proliferation and differentiation of intestinal epithelial cell models by directing a broad gene expression program that includes SOX9 and the ubiquitin ligase pathway. *J. Biol. Chem.* 291 15975–15984. 10.1074/jbc.m116.733733 27261459 PMC4965549

[B92] Pierdomenico M. Cesi V. Cucchiara S. Vitali R. Prete E. Costanzo M. (2016). NOD2 is regulated by miR-320 in physiological conditions but this control is altered in inflamed tissues of patients with inflammatory bowel disease. *Inflamm. Bowel Dis.* 22 315–326. 10.1097/mib.0000000000000659 26752466

[B93] Quigley E. M. M. Gajula P. (2020). Recent advances in modulating the microbiome. *F1000Res.* 9:F1000FacultyRev–46. 10.12688/f1000research.20204.1 PMC6993818 32047611

[B94] Rodríguez-Nogales A. Algieri F. Garrido-Mesa J. Vezza T. Utrilla M. P. Chueca N. (2018a). Intestinal anti-inflammatory effect of the probiotic *Saccharomyces boulardii* in DSS-induced colitis in mice: Impact on microRNAs expression and gut microbiota composition. *J. Nutr. Biochem.* 61 129–139. 10.1016/j.jnutbio.2018.08.005 30236870

[B95] Rodríguez-Nogales A. Algieri F. Garrido-Mesa J. Vezza T. Utrilla M. P. Chueca N. (2018b). The administration of *Escherichia coli Nissle* 1917 ameliorates development of DSS-induced colitis in mice. *Front. Pharmacol.* 9:468. 10.3389/fphar.2018.00468 29867475 PMC5958303

[B96] Rooks M. G. Garrett W. S. (2016). Gut microbiota, metabolites and host immunity. *Nat. Rev. Immunol.* 16 341–352. 10.1038/nri.2016.42 27231050 PMC5541232

[B97] Rowland I. Gibson G. Heinken A. Scott K. Swann J. Thiele I. (2018). Gut microbiota functions: metabolism of nutrients and other food components. *Eur. J. Nutr.* 57 1–24. 10.1007/s00394-017-1445-8 28393285 PMC5847071

[B98] Rupaimoole R. Slack F. J. (2017). MicroRNA therapeutics: towards a new era for the management of cancer and other diseases. *Nat. Rev. Drug Discov.* 16 203–222. 10.1038/nrd.2016.246 28209991

[B99] Saliminejad K. Khorram Khorshid H. R. Ghaffari S. H. (2019). Why have microRNA biomarkers not been translated from bench to clinic? *Fut. Oncol.* 15 801–803. 10.2217/fon-2018-0812 30652506

[B100] Salvucci E. (2019). The human-microbiome superorganism and its modulation to restore health. *Int. J. Food Sci. Nutr.* 70 781–795. 10.1080/09637486.2019.1580682 30843443

[B101] Sanders M. E. Merenstein D. J. Reid G. Gibson G. R. Rastall R. A. (2019). Probiotics and prebiotics in intestinal health and disease: from biology to the clinic. *Nat. Rev. Gastroenterol. Hepatol.* 16 605–616. 10.1038/s41575-019-0173-3 31296969

[B102] Sarshar M. Scribano D. Ambrosi C. Palamara A. T. Masotti A. (2020). Fecal microRNAs as innovative biomarkers of intestinal diseases and effective players in host-microbiome interactions. *Cancers* 12:E2174. 10.3390/cancers12082174 PMC7463924 32764361

[B103] Schönauen K. Le N. von Arnim U. Schulz C. Malfertheiner P. Link A. (2018). Circulating and fecal microRNAs as biomarkers for inflammatory bowel diseases. *Inflamm. Bowel Dis.* 24 1547–1557. 10.1093/ibd/izy046 29668922

[B104] Sehrawat N. Yadav M. Singh M. Kumar V. Sharma V. R. Sharma A. K. (2020). Probiotics in microbiome ecological balance providing a therapeutic window against cancer. *Semin. Cancer Biol.* 20 S1044–S1579. 10.1016/j.semcancer.2020.06.009 32574811

[B105] Shirafkan N. Mansoori B. Mohammadi A. Shomali N. Ghasbi M. Baradaran B. (2018). MicroRNAs as novel biomarkers for colorectal cancer: new outlooks. *Biomed. Pharmacother.* 97 1319–1330. 10.1016/j.biopha.2017.11.046 29156521

[B106] Singh N. Shirdel E. A. Waldron L. Zhang R. H. Jurisica I. Comelli E. M. (2012). The murine caecal microRNA signature depends on the presence of the endogenous microbiota. *Int. J. Biol. Sci.* 8 171–186. 10.7150/ijbs.8.171 22211115 PMC3248702

[B107] Soroosh A. Koutsioumpa M. Pothoulakis C. Iliopoulos D. (2018). Functional role and therapeutic targeting of microRNAs in inflammatory bowel disease. *Am. J. Physiol. Gastrointest Liver Physiol.* 314 G256–G262. 29146677 10.1152/ajpgi.00268.2017 PMC5866423

[B108] Spinler J. K. Karri V. Hirschi K. D. (2019). Planting the microbiome. *Trends Microbiol.* 27 90–93. 10.1016/j.tim.2018.12.001 30600139

[B109] Suez J. Zmora N. Segal E. Elinav E. (2019). The pros, cons, and many unknowns of probiotics. *Nat. Med.* 25 716–729. 10.1038/s41591-019-0439-x 31061539

[B110] Tan S. Shi H. Ba M. Lin S. Tang H. Zeng X. (2016). miR-409-3p sensitizes colon cancer cells to oxaliplatin by inhibiting Beclin-1-mediated autophagy. *Int. J. Mol. Med.* 37 1030–1038. 10.3892/ijmm.2016.2492 26935807

[B111] Tang L. (2019). Diet influences microbe-host interaction. *Nat. Methods.* 16:361. 10.1038/s41592-019-0413-z 31040430

[B112] Tarallo S. Ferrero G. Gallo G. Francavilla A. Clerico G. Realis Luc A. (2019). Altered fecal small RNA profiles in colorectal cancer reflect gut microbiome composition in stool samples. *mSystems* 4 e289–e219. 10.1128/mSystems.00289-19 PMC6749105 31530647

[B113] Teng Y. Ren Y. Sayed M. Hu X. Lei C. Kumar A. (2018). Plant-derived exosomal microRNAs shape the gut microbiota. *Cell Host. Microbe.* 24 637–652.e. 30449315 10.1016/j.chom.2018.10.001 PMC6746408

[B114] Tian Y. Xu J. Li Y. Zhao R. Du S. Lv C. (2019). MicroRNA-31 reduces inflammatory signaling and promotes regeneration in colon epithelium, and delivery of mimics in microspheres reduces colitis in mice. *Gastroenterology* 156 2281–2296.e. 30779922 10.1053/j.gastro.2019.02.023

[B115] Tili E. Michaille J. J. Piurowski V. Rigot B. Croce C. M. (2017). MicroRNAs in intestinal barrier function, inflammatory bowel disease and related cancers-their effects and therapeutic potentials. *Curr. Opin. Pharmacol.* 37 142–150. 10.1016/j.coph.2017.10.010 29154194 PMC5938753

[B116] To K. K. Tong C. W. Wu M. Cho W. C. (2018). MicroRNAs in the prognosis and therapy of colorectal cancer: From bench to bedside. *World J. Gastroenterol.* 24 2949–2973. 10.3748/wjg.v24.i27.2949 30038463 PMC6054943

[B117] Turchinovich A. Weiz L. Langheinz A. Burwinkel B. (2011). Characterization of extracellular circulating microRNA. *Nucleic Acids Res.* 39 7223–7233. 10.1093/nar/gkr254 21609964 PMC3167594

[B118] Virtue A. T. McCright S. J. Wright J. M. Jimenez M. T. Mowel W. K. Kotzin J. J. (2019). The gut microbiota regulates white adipose tissue inflammation and obesity via a family of microRNAs. *Sci. Transl. Med.* 11:eaav1892. 10.1126/scitranslmed.aav1892 31189717 PMC7050429

[B119] Wan M. L. Y. Forsythe S. J. El-Nezami H. (2019). Probiotics interaction with foodborne pathogens: a potential alternative to antibiotics and future challenges. *Crit. Rev. Food Sci. Nutr.* 59 3320–3333. 10.1080/10408398.2018.1490885 29993263

[B120] Wang F. Liu J. Zou Y. Jiao Y. Huang Y. Fan L. (2017). MicroRNA-143-3p, up-regulated in *H. pylori*-positive gastric cancer, suppresses tumor growth, migration and invasion by directly targeting AKT2. *Oncotarget* 8 28711–28724. 10.18632/oncotarget.15646 28404925 PMC5438685

[B121] Wang H. Chao K. Ng S. C. Bai A. H. Yu Q. Yu J. (2016). Pro-inflammatory miR-223 mediates the cross-talk between the IL23 pathway and the intestinal barrier in inflammatory bowel disease. *Genome Biol.* 17:58. 10.1186/s13059-016-0901-8 PMC4815271 27029486

[B122] Wang H. Peng R. Wang J. Qin Z. Xue L. (2018). Circulating microRNAs as potential cancer biomarkers: the advantage and disadvantage. *Clin. Epigenet.* 10:59. 10.1186/s13148-018-0492-1 PMC5913875 29713393

[B123] Weber J. A. Baxter D. H. Zhang S. Huang D. Y. Huang K. H. Lee M. J. (2010). The microRNA spectrum in 12 body fluids. *Clin. Chem.* 56 1733–1741. 10.1373/clinchem.2010.147405 20847327 PMC4846276

[B124] Wu C. W. Ng S. C. Dong Y. Tian L. Ng S. S. Leung W. W. (2014). Identification of microRNA-135b in stool as a potential noninvasive biomarker for colorectal cancer and adenoma. *Clin. Cancer Res.* 20 2994–3002. 10.1158/1078-0432.ccr-13-1750 24691020

[B125] Xing S. C. Xing S. C. Huang C. B. Mi J. D. Wu Y. B. Liao X. D. (2019). *Bacillus coagulans* R11 maintained intestinal villus health and decreased intestinal injury in lead-exposed mice by regulating the intestinal microbiota and influenced the function of faecal microRNAs. *Environ. Pollut.* 255:113139. 10.1016/j.envpol.2019.113139 31563774

[B126] Xue X. Feng T. Yao S. Wolf K. J. Liu C. G. Liu X. (2011). Microbiota downregulates dendritic cell expression of miR-10a, which targets IL-12/IL-23p40. *J. Immunol.* 187 5879–5886. 10.4049/jimmunol.1100535 22068236 PMC3226774

[B127] Yadav M. Pratyoosh S. (2019). Recent systems biology approaches for probiotics use in health aspects: a review. *3 Biotech.* 9:448. 10.1007/s13205-019-1980-5 PMC6848287 31763126

[B128] Yamamoto H. Mori M. (2016). MicroRNAs as therapeutic targets and colorectal cancer therapeutics. *Adv. Exp. Med. Biol.* 937 239–247. 10.1007/978-3-319-42059-2_13 27573904

[B129] Yan F. Polk D. B. (2020). Probiotics and probiotic-derived functional factors-mechanistic insights into applications for intestinal homeostasis. *Front. Immunol.* 11:1428. 10.3389/fimmu.2020.01428 32719681 PMC7348054

[B130] Yang Y. Ma Y. Shi C. Chen H. Zhang H. Chen N. (2013). Overexpression of miR-21 in patients with ulcerative colitis impairs intestinal epithelial barrier function through targeting the Rho GTPase RhoB. *Biochem. Biophys. Res. Commun.* 434 746–752. 10.1016/j.bbrc.2013.03.122 23583411

[B131] Yousefi B. Eslami M. Ghasemian A. Kokhaei P. Salek Farrokhi A. Darabi N. (2019). Probiotics importance and their immunomodulatory properties. *J. Cell Physiol.* 234 8008–8018. 10.1002/jcp.27559 30317594

[B132] Yu X. Odenthal M. Fries J. W. (2016). Exosomes as miRNA carriers: formation-function-future. *Int. J. Mol. Sci.* 17:2028. 10.3390/ijms17122028 27918449 PMC5187828

[B133] Yuan C. Burns M. B. Subramanian S. Blekhman R. (2018). Interaction between host microRNAs and the gut microbiota in colorectal cancer. *mSystems* 3 e205–e217. 10.1128/mSystems.00205-17 PMC5954203 29795787

[B134] Zeng Z. Li Y. Pan Y. Lan X. Song F. Sun J. (2018). Cancer-derived exosomal miR-25-3p promotes pre-metastatic niche formation by inducing vascular permeability and angiogenesis. *Nat. Commun.* 9:5395. 10.1038/s41467-018-07810-w PMC6300604 30568162

[B135] Zhang C. X. Wang H. Y. Chen T. X. (2019). Interactions between intestinal microflora/probiotics and the immune system. *Biomed. Res. Int.* 2019:67 64919. 10.1155/2019/6764919 PMC6886316 31828119

[B136] Zhou B. Song J. Han T. Huang M. Jiang H. Qiao H. (2016). MiR-382 inhibits cell growth and invasion by targeting NR2F2 in colorectal cancer. *Mol. Carcinog.* 55 2260–2267. 10.1002/mc.22466 26800338

[B137] Zhou G. Zhou Y. Chen X. (2017). New insight into inter-kingdom communication: horizontal transfer of mobile small RNAs. *Front. Microbiol.* 8:768. 10.3389/fmicb.2017.00768 28507539 PMC5410588

[B138] Zhou M. He J. Shen Y. Zhang C. Wang J. Chen Y. (2017). New frontiers in genetics, gut microbiota, and immunity: a rosetta stone for the pathogenesis of inflammatory bowel disease. *Biomed. Res. Int.* 2017:8201672. 10.1155/2017/8201672 PMC5558637 28831399

[B139] Zhou Q. Zhu Y. Wei X. Zhou J. Chang L. Sui H. (2016). MiR-590-5p inhibits colorectal cancer angiogenesis and metastasis by regulating nuclear factor 90/vascular endothelial growth factor A axis. *Cell Death Dis.* 7:e2413. 10.1038/cddis.2016.306 27735951 PMC5133975

[B140] Zimmermann M. Zimmermann-Kogadeeva M. Wegmann R. (2019). Mapping human microbiome drug metabolism by gut bacteria and their genes. *Nature* 570 462–467. 10.1038/s41586-019-1291-3 31158845 PMC6597290

[B141] Zou L. Xiong X. Wang K. Yin Y. (2018). MicroRNAs in the intestine: role in renewal, homeostasis, and inflammation. *Curr. Mol. Med.* 18 190–198. 10.2174/1566524018666180907163638 30198431

